# Rare Case of Multifocal Alveolar Echinococcosis

**DOI:** 10.5334/jbsr.3467

**Published:** 2024-03-14

**Authors:** Sarah Van Steenberge, Axel Boyer, Romain Pierre Gillard

**Affiliations:** 1 Intern in Gastroenterology. Gastroenterology Departement. CHU Sart-Tilman Belgium; 2 Intern in Radiology. Radiology Department. CHR Citadelle Belgium; 3 Head of Departement. Radiology Department. CHU Sart-Tilman Belgium

**Keywords:** Scattered calcifications, tumor-like parasitosis, echinococcosis, CT, MRI

## Abstract

*Teaching Point:* Hepatic alveolar echinococcosis can mimic a slow-growing tumor, and multi-organ involvement is rare; imaging has a crucial role in diagnosing this zoonosis that is endemic in the southern part of Belgium.

## Case History

**Figure 1:**
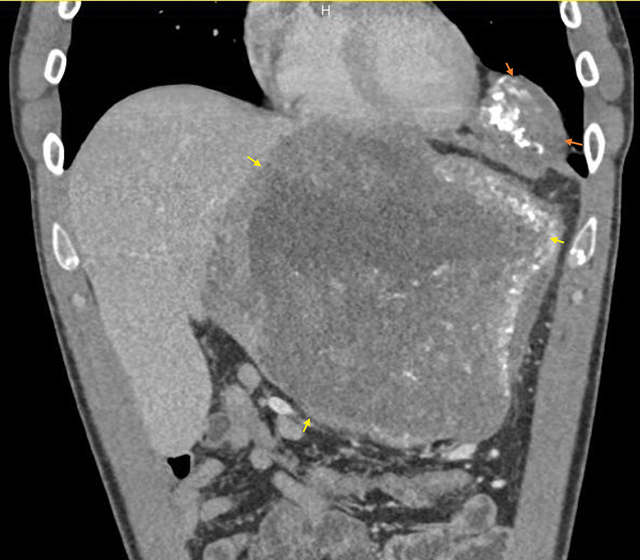
Portal CT coronal plane. Left hepatic (yellow arrows) and left cardiophrenic masses (orange arrows) with calcifications.

A 32-year-old male presented to the emergency unit with epigastralgia and weight loss over the past year. Abdominal computed tomography (CT) in the portal phase depicted a diffuse involvement of the left hepatic lobe by a confluent necrotic mass with irregular margins and peripheral scattered calcifications (Figure 1—yellow arrow).

A second location of 8 cm was present in the left cardiophrenic recess (Figure 1—orange arrow) and a third mediastinal (not shown). This pattern is compatible with hepatic alveolar echinococcosis.

**Figure 2:**
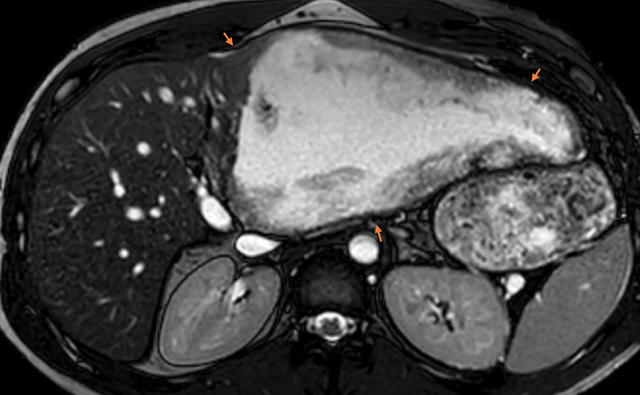
Axial T2 TSE MRI. The left hepatic mass (orange arrows) has a necrotic content (hyperT2) with peripheric fibrosis (hypoT2) and microcyst (hyperT2).

A magnetic resonance imaging (MRI) was performed, showing hyperT2 content (necrosis) with multiple peripheral microcystic lesions. Hepatic mass contours are fibrous with marked T2 hyposignal (Figure 2—orange arrow) and without any enhancement.

**Figure 3:**
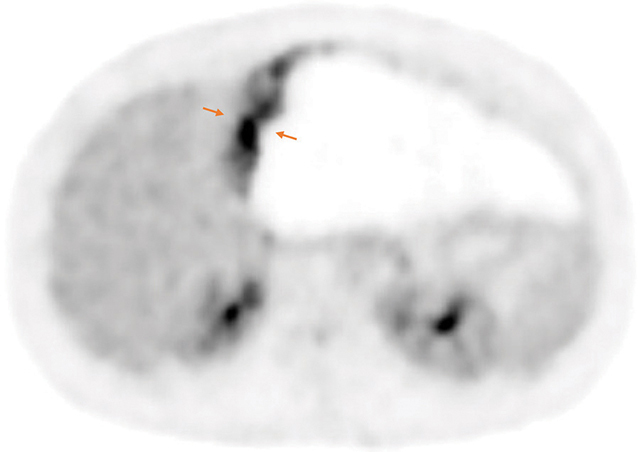
Axial 18FDG PET-CT. High 18FDG uptake in the left lesion part (orange arrow).

The positron emission tomography (PET)–CT showed a periphery 18FDG fixation after 3 hours, indicating intense late metabolic activity (Figure 3—orange arrow).

They are suggestive of alveolar echinococcosis, especially in the context of occupational exposure.

A left hepatectomy and histological examination confirmed the diagnosis.

The patient was treated with Albendazole indefinitely; the mediastinal and cardiophrenic lesions were not resected due to potential major complications.

## Comment

Echinococcosis is a cosmopolitan zoonosis caused by the larval stage of a plathelminth of the cestode class, known as Echinococcus. The definitive natural host of this parasite is the red fox (Vulpes vulpes). Up to 50% of southern Belgium foxes are estimated carriers of this parasite.

Two species can infect humans, with different presentations: *Echinococcus granulosus* causes the cystic form (hydatid cyst), while *Echinococcus multilocularis* is responsible for the alveolar form. The alveolar form has the particularity of developing slowly in the liver parenchyma, like a neoplastic disease, and can be responsible for distant lesions by hematogenous dissemination.

In 99% of cases, the primary site of infection is the liver; two or more may be affected organs in 15% to 20% of cases (including lung, heart, brain, and bones) [1].

The diagnosis is based on radiological imaging combined with biological tests (hypereosinophilia, ELISA, and Western Blot serologies) and polymerase chain reaction (PCR) analysis of tissue samples.

Without treatment, the disease is fatal within 10 years after the onset of symptoms. The curative treatment is medical and surgical and ideally involves a partial segmental hepatectomy after assessment of the extension, combined with 2 years of anti-parasitic therapy. Patients are then monitored for 10 years.
